# Low-Dimensional Materials and State-of-the-Art Architectures for Infrared Photodetection

**DOI:** 10.3390/s18124163

**Published:** 2018-11-27

**Authors:** Nasir Ilyas, Dongyang Li, Yuhao Song, Hao Zhong, Yadong Jiang, Wei Li

**Affiliations:** 1 School of Optoelectronic Information, University of Electronic Science and Technology of China, Chengdu 610054, China; ilyas_nasir84@yahoo.com (N.I.); open_please@163.com (D.L.); 15528122306@163.com (Y.S.); zhonghao729@foxmail.com (H.Z.); 2 State Key Laboratory of Electronic Thin Films and Integrated Devices, University of Electronic Science and Technology of China, Chengdu 610054, China; jiangyd@uestc.edu.cn

**Keywords:** infrared photodetectors, plasmonic waveguides, nanostructures

## Abstract

Infrared photodetectors are gaining remarkable interest due to their widespread civil and military applications. Low-dimensional materials such as quantum dots, nanowires, and two-dimensional nanolayers are extensively employed for detecting ultraviolet to infrared lights. Moreover, in conjunction with plasmonic nanostructures and plasmonic waveguides, they exhibit appealing performance for practical applications, including sub-wavelength photon confinement, high response time, and functionalities. In this review, we have discussed recent advances and challenges in the prospective infrared photodetectors fabricated by low-dimensional nanostructured materials. In general, this review systematically summarizes the state-of-the-art device architectures, major developments, and future trends in infrared photodetection.

## 1. Introduction

**Figure 1:**
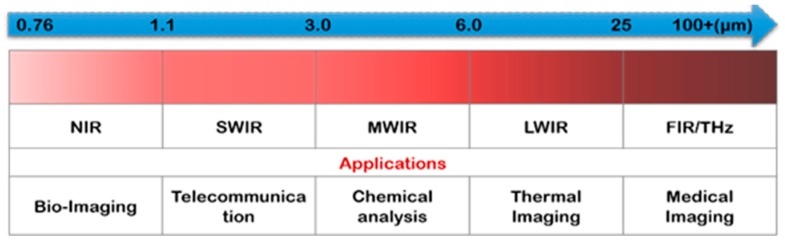
The typical photodetector applications at broad infrared detection spectral bands, including NIR, SWIR, MWIR, LWIR and FIR/THz.

The extensive study of light–matter interaction has unveiled many dark secrets of the universe and has led to many revolutionary scientific discoveries. The interaction of light with semiconductors has particularly enabled us to understand the behavior of various fundamental phenomena and laid the foundation of optoelectronic systems that we rely on today. Most of these systems involve the detection of light, and most of them are commonly found in our routine life such as digital cameras and night vision goggles [1]. The rapid advancement in semiconductor materials in the past few decades have developed photodetectors for various applications such as motion detection, telecommunication, astronomy observation [2], and atmospheric spectroscopy [3]. It involves the detection of light with broad-range wavelengths from 0.75 to ≥1000 µm, covering the spectrum of near-IR (NIR), short-wavelength IR (SWIR), mid-wavelength IR (SWIR), long- wavelength IR (LWIR), and far-IR (FIR). Figure 1 shows some typical applications of IR-photodetectors from NIR to FIR spectra.

Over the last few years, research and development efforts at the nanoscale interaction of light with materials have enabled researchers to develop highly efficient photodetectors responsive to a particular region of the electromagnetic (EM) spectrum [4,5,6,7]. The reduced geometry impacts the response time up to ~picosecond, which leads to facilitation of the fabrication of high-performance photodetectors for optical communication and high frame cameras [8,9]. In last few years, lead sulfide (PbS) nanocrystal has emerged as one of the most relatively substantive materials for infrared photodetectors (IRPDs). The PbS-based photodetectors are advantageous in various aspects, including low-cost manufacturing, wideband sensitivity, and flexible substrate compatibility [4]. Photodetectors assembled with PbS quantum dots and 2D graphene have reached an ultra-high detectivity over 7 × 10^13^ Jones, with exceptionally high photo gain (≥10^8^) @ MWIR band [10].

The p–n or Schottky junction is a building block of the various modern semiconductor devices. By developing p–n or the Schottky junction in nanowire, including different hybrid heterostructure-based infrared photodetectors, a low-leakage current has been achieved [11,12,13,14,15]. Moreover, researchers have successfully suppressed dark current and shown prospective in constructing room temperature infrared photodetectors (IRPDs) with enhanced optoelectronic properties. The low-dimensional nanostructured materials have added some additional functionalities such as color- and polarization-sensitive detection in existing photodetector devices [16,17,18,19,20]. Furthermore, the manipulation of light with nanostructured materials beyond the diffraction limit significantly enhances at the sub-wavelength level [21,22,23,24,25]. In nanostructured materials, several achievements from different aspects such as detectivity, response speed, and operational temperature have been pointed out for high-performance IRPDs.

A material dubbed black silicon (BS) has shown great promise for making cheaper, more sensitive light detectors and imaging devices. Due to its nanostructured surface and narrow band gap, it is feasible to achieve high absorptance (>90%) over a broad spectrum from 250 nm to 2500 nm [26]. Additionally, the high photoconductive gain and responsivity (0.57 A/W at 1050 nm wavelength) [27] gives rise to the practical device applications for IR detection.

Despite traditional low-dimensional materials, exploring novel materials with rapidly controlled size and advanced architectures are crucial to improving the performance and functionalities of the existing devices. Therefore, this article discusses the recent developments and trends in state-of-the-art IRPDs based on novel low-dimensional nanostructured materials such as quantum wells (QWs), quantum dots (QDs), nanowires (NWs), and 2D materials including graphene and black phosphorus. Moreover, some hybrid combinations of different low-dimensional materials and integration of plasmonic nanostructures for the confinement of light beyond the diffraction limits are also discussed. Furthermore, we will summarize the possible challenges and prospects of these photodetectors.

## 2. Nanostructured IR Sensitive Materials for Photodetectors

Nanostructured IR sensitive materials have gained significant interests in photodetection technology. The enhancement of photosensitivity becomes possible by tailoring the materials’ properties and their geometry. In this section, we will discuss the current advancements in IRPDs based on different nanostructured materials and the related architectures such as quantum wells (QWs), quantum dots (QDs), nanowires (NWs), and 2D layers and their hybrid heterostructures (HSs). Table 1 gives the IRPDs based on various nanostructured materials with their primary performance parameter.

### 2.1. Quantum Dots and Quantum Wells-Based Photodetectors

**Figure 2:**
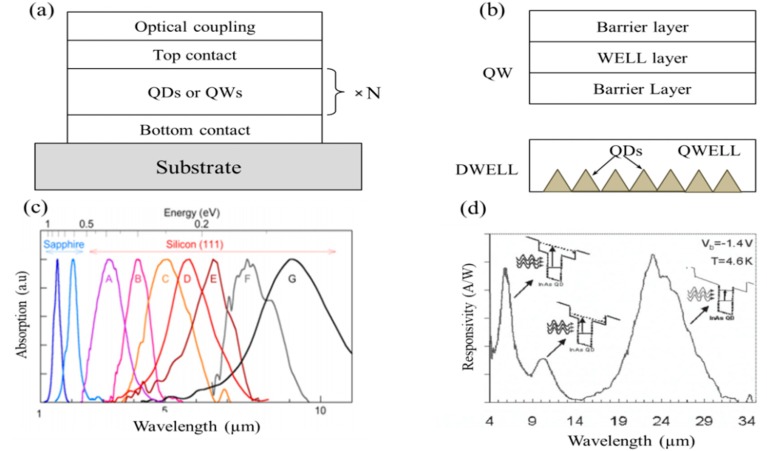
(**a**) Schematic of QW or QD layers with optical coupling layer and (**b**) structures of sandwiched QWs with multiple barrier layers and dot-in-well (QWELL) structure, indicated below (**c**) The tunable absorption in GaN/AlGaN QWs is shown by adjusting the width of the GaN QW layer (reproduced with the permission of Reference [49], Copyright 2009, IOP Publishing). Moreover, (**d**) shows the multiple band spectral response in a InAs/InGaAs/GaAs dot-in-well structure, (Reproduced with the permission of Reference [50]. Copyright 2007, IEEE).

Initially, QW and QD-based IRPDs were considered as the replacements for conventional Ge, HgCdTe, and related alloys in the mid-waves IR band, realizing their prospects in relatively high operating temperature and chip integration [31,42,43]. In practical, quantum wells and dots are defined in sandwiched structures with a well or dot layer capped in barrier layers of conduction-band mismatched materials through epitaxial growth methods (i.e., MOCVD, MBE) as shown in Figure 2a [44,45]. The photo-excited electrons come across the energy barrier which formed between the interface of well or dot layers and barrier layers due to mismatched energy bands. The transition of photo-excited electrons across the interface of well or dot layers and barrier layers enable the detection of light beyond the bandgap limit of the material. Therefore, the broadband IR photodetection in the range of SWIR to FIR have been achieved by tailoring the geometry of such epitaxially grown QWs and dots, and by modifying the interfacial surface chemistry of barriers layers. For efficient light coupling in practical photodetector devices, grooved or surface grating layers on absorption layers have been used [46]. After the invention of the first QD-based photodetectors, several successes have since been made in performance and functionalities [46,47]. By using advanced growth techniques and dopant incorporation, different photodetector designs (i.e., dot-in-well (DWELL), quantum cascade) have been brought in to optimize the photodetection performances [47,48]. Figure 2b shows typical QW and dot-in-well (DWELL) structures. The enhanced optoelectronic properties and corresponding performance parameters can be achieved by merely tailoring the geometry of either QD or QW structures in these photodetectors. The broad spectral response of quantized GaN/AlGaN superlattice type-II and InAs/InGaAs/GaAs DWELL systems is shown in Figure 2c,d. Moreover, the broad absorption spectral response can also be realized by tailoring the width of the GaN QW layer and in DWELL structure barrier layers resulting in the multiband photo-response spectra [49,50]. The multiband photo-response in DWELL structures makes possible the construction of multi-color sensitive photodetectors for thermal image processing.

However, for a successful intraband transition in QWs and QDs for IR detection, heavily-doped semiconductor materials have been used for populating the carriers in the ground state of the desired energy band. Due to such heavy doping, the photodetectors undergo high dark currents, which is undesirable for practical device application and integration of FPAs with readout circuits. Therefore, resonance tunneling barrier layers have been introduced to control the dark and thermally-produced current carriers [51,52]. Moreover, quantum cascade detectors (QCDs) have already been proposed for passive IRPDs. In these photodetectors, asymmetric energy band alignment is used for the mid-wave IR region as well as for even longer wave IR detection [30,53,54,55,56].

Rapid development in colloidal quantum dots (CQDs) like PbS [57,58,59], PbSe [60,61], Ag_2_Se [62], HgTe [63,64], and HgSe [20] is providing the promise of an alternative to epitaxially grown QD-based IRPDs in various aspects, such as compatibility with a variety of substrates and cost-effective processing. The typical strong quantum confinement in CQDs has provided unique optoelectronic properties such as ultra-high photoconductive gain achieved through phonons bottleneck effect and surface traps [65,66,67]. The extensive research efforts in various CQDs synthesis methodologies to define their geometrical structures, stoichiometric composition, and their surface chemistry have improved their optical response span in IR spectra with high electrical performance [68]. The CQD-based photodetectors have high photoconductivity gain as compared to their epitaxially grown counterparts [35,69,70]. Recently, a remarkably high detectivity of ~1.8 × 10^13^ Jones at 1.3 µm has been reported in PbS CQD-based photodetectors [35]. Most importantly, a unique feature, “multiexciton generation”, during light absorption has been explored in CQD-based photodetectors [71,72], which has resulted in optical gain efficiency more than unity [73,74].

**Figure 3:**
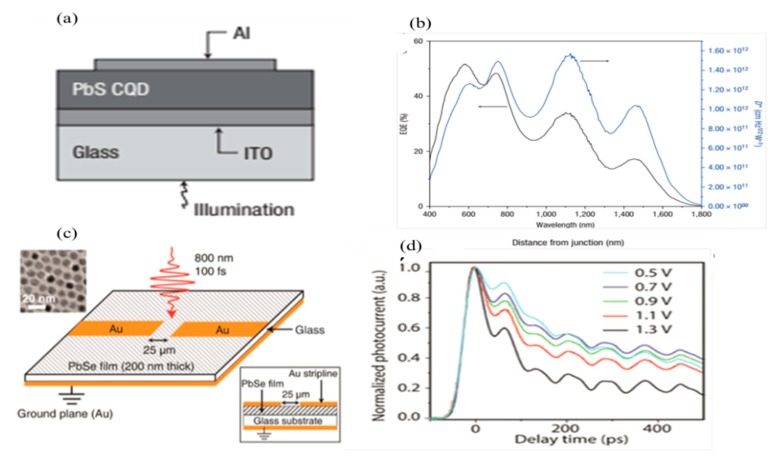
(**a**) The structure and (**b**) quantum efficiency of the Schottky diode Al/PbS CQDs of a Schottky diode photodetector based on a PbS CQD film (reproduced with the permission of Reference [69] Copyright 2009, Nature Publishing Group). (**c**) The structure of a vertically stacked photoconductive detector based on PbSe CQDs and (**d**) the response time at a different applied voltage (reproduced with the permission of Reference [75], Copyright 2016, American Chemical Society).

Despite their remarkable advantages, performance related issues like poor response time still need serious considerations. It is well known that the native surface oxides result in high detectivity, while at the same time these oxides limit response speed. Therefore, different strategies have been employed to avoid this issue. In the device (Figure 3a), a 350 nm thin film of PbS CQDs is sandwiched between ITO and Al electrodes. A depletion region is formed in the device near the PbS/Al contact due to the presence of Schottky barrier and remaining part is considered as quasi-neutral region [69]. The carriers are separated due to drift/diffusion mechanisms in both the depletion and quasi-neutral region resulted in an acceptable response time with high detectivity. The photoresponsivity depends on the width of the Schottky barrier, and it could be varied by applying an externally biased voltage.

In the fully depleted region, a significant photo-response was achieved up to a 3-dB frequency over a wavelength of ~1.5 µm, while the detectivity was still over 10^12^ Jones for this device (Figure 3b). The photo-generated carriers’ dynamic and response time in nanoseconds was achieved by applying an external bias voltage. Accordingly, ultrafast current dynamics was accomplished in the ITO/PbSe structure with CQDs/Au contact as shown in Figure 3d. By applying the externally biased voltage, the photo-generated carriers were separated before trapping with the highest response time at 1.3 V (Figure 3d). In the meantime, to avoid a risk of high dark current, multiple blocking layers were required for performance optimization [76]. The thermal imaging in the mid-wave IR band (3–5 µm) and longer waves IR band (8–12 µm) was predominantly carried out with traditional HgCdTe or InAsSb-based focal plane arrays (FPAs).

**Figure 4:**
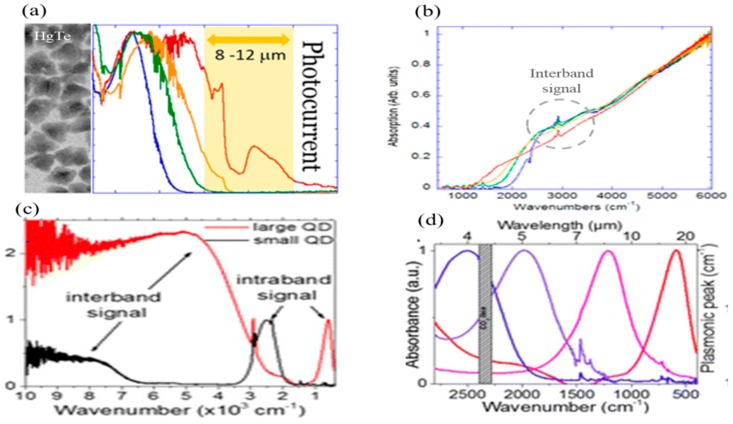
(**a**) TEM image of HgTe CQDs with broadband photo-response; (**b**) shows the electron transitions for corresponding wavenumbers (reproduced with the permission of Reference [75], Copyright 2014, American Chemical Society); (**c**) The intraband transition spectra and (**d**) absorption spectra in HgSe CQD-based photodetectors (reproduced with the permission of Reference [20], Copyright 2016, American Chemical Society).

Recently, the CQDs of low band gap materials such as HgTe [77] and HgSe [20,78] have also been synthesized. Cut-off wavelengths have been achieved up to 12 µm in HgTe CQD-based IRPDs [75,79]. Figure 4a,b show the size and absorption spectra of HgTe CQDs in the range of 15 nm to 20 nm, and an absorption gap of 5 µm to 8 µm wavelength at room temperature. Moreover, the absorption in the longer wavelength of ~8–12 µm can also be achieved by cooling to liquid nitrogen temperature. Recently, HgSe CQD-based photodetectors have successfully fabricated for NIR to the THz range with significantly extended absorption edges up to 20 µm at room temperature [20]. From Figure 4c, it is clear that typical intraband transition appears between 500~3000 cm^−1^. The sharp peaks in IR absorption bands in HgSe CQDs indicate discrete quantum states, while the continuously tunable behavior in the range of 3 µm to 20 µm wavelength was observed by varying the size of CQDs. Therefore, practically assembled photodetectors based on CQDs has shown promising photodetection performance over 1 × 10^8^ Jones at of ~6 µm wavelength (Figure 4d).

### 2.2. Nanowires

One-dimensional (1D) nanostructures such as NWs and NTs have attracted significant research interest in the field of optoelectronic devices (i.e., lasers, photodetectors) [80]. The photoconductive gain mechanism in 1D and CQD structures is the same due to their similar surface to volume ratio. The inherent anisotropic nature of nanowires renders fast carrier mobility and the feasibility in constructing polarization sensitive photodetectors. The III-V semiconductor NWs have attracted considerable research interest due to their direct bandgap and high carrier mobilities [81,82,83]. In addition to conventional semiconductor nanowires, the polymer-based optical nanowires attract a significant research interest considering importance in optical applications and functionalities (e.g., optical intensity, wavelength, polarization, phase, and fluorescence) [84,85,86]. The protein-based single nanowires and nanowire devices could be readily fabricated by using femtosecond laser direct writing (FLDW) [87,88,89,90,91,92]. Recently, the one optical window of the protein single nanowire waveguides at ≈680 nm coinciding with the red or near-IR biological window has been reported [93,94]. The significant merits, such as versatility, facilely adjustable high sensitivity, biocompatibility, satisfactory designability, and repeatability of nanowire-devices may endow the all-protein-based single nanowire biosensors with potential applications in bio-X environments.

**Figure 5:**
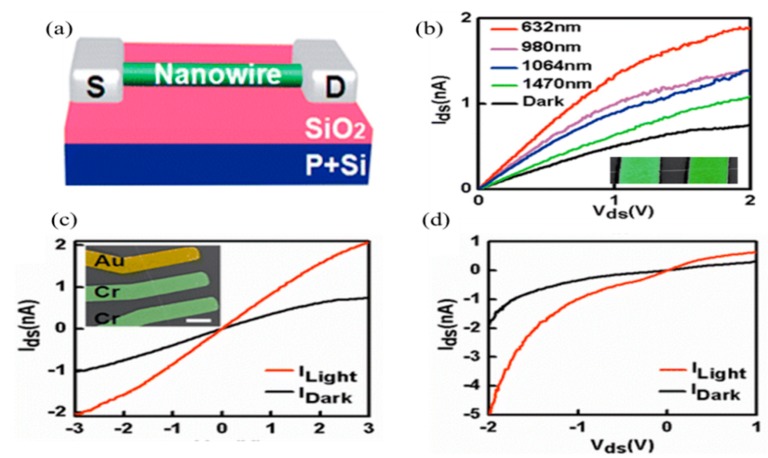
(**a**) Schematic illustration of an InAs NW-based photodetector; (**b**) photocurrent spectra of an InAs NW near-infrared photodetector changing from red to NIR light and the inset is the SEM image of a representative photodetector; (**c**) the dark and photocurrent at ohmic–ohmic combinations in the detectors and the inset shows Au/Cr contact as electrodes; (**d**) the photo-response at the Schottky–Ohmic contact combination (reproduced with the permission of Reference [37], Copyright 2014, American Chemical Society).

Recently, ultra-high photocurrent gain was reported in GaN NW-based photodetectors at room temperature, which is three times higher than those for their thin-film counterparts. Furthermore, ultra-high photo-response (~ps) in mid-wave IR with high mobility at room temperature was reported in core-shell NWs (e.g., InGaN InAs/InP, InAs/InAsSb) based photodetectors [95,96,97,98]. In Figure 5c, along with the same InAs NW, an Ohmic–Ohmic contact was fabricated using Cr/Au as electrodes, due to different work functions as a Schottky barrier achieved at the contact interface. As shown in the Figure 5c,d, the dark and photocurrent measurements with the aforementioned Ohmic and Schottky barrier combinations in the device, respectively, whereas in the Schottky–Ohmic contact, a depletion region developed in the InAs NW, resulting in a significant decrease in dark current. Also, the built-in electrical field at contact interface also enhances the charge separation efficiency. Therefore, in InAs nanowire-based photoconductors, a high photo-response up to 5.3 × 10^3^ AW^−1^ was achieved at 1.5 µm by adopting the Schottky contacts.

**Figure 6:**
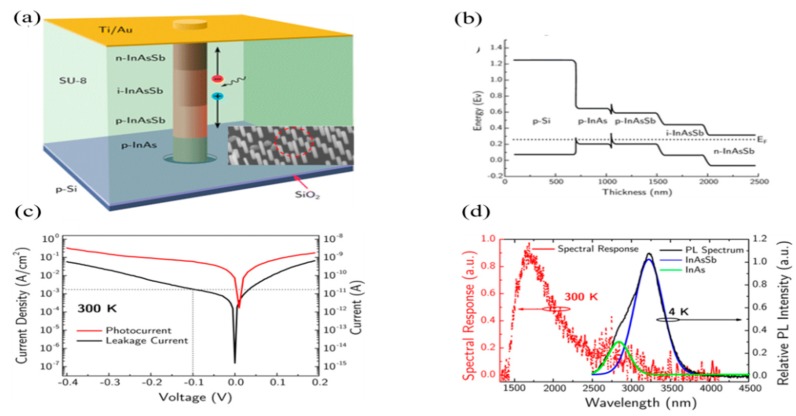
(**a**) The schematic of InAs_1-x_ Sb_x_ nanowire with p–i–n junction vertically and the inset is the SEM image of NWs array on a p–Si substrate; (**b**) shows the cascade-like energy band alignments along the NW at different stichometry (x) values; (**c**) dark and photocurrents corresponding to 1.55 µm infrared light, and (**d**) the photo-response at different temperature in an InAs_1-x_ Sb_x_-based photodetector. (Reproduced with the permission of Reference [11]. Copyright 2016, American Chemical Society).

Besides Schottky contacts, dopant gradient mechanism in NWs were also used to improve the on–off ratio and suppress the dark current in III-V NW-based IRPDs [11,12,83]. Figure 6a shows a schematic of InAsSb NW-based photodetectors. The p–i–n type junction was realized by dopant incorporation during the vapor phase growth of NWs. The cascade-like energy band alignment in InAs_1-x_Sb_x_ NW formed along the dopant gradient direction (shown in Figure 6b). The built-in p–i–n junction in photodetectors works like a photovoltaic mode, which possesses the negligible risk of dark current (Figure 6c). The most extended wavelength detection of ~2.5 µm was reported in InAs_1-x_Sb_x_ NW-based photodetectors at room temperature (Figure 6d). Here, the photogate effect took place by the accumulation of a carrier on the InAs NW surface due to native oxides, which significantly improved the carrier mobility and detectivity [17,18,19,20].

**Figure 7:**
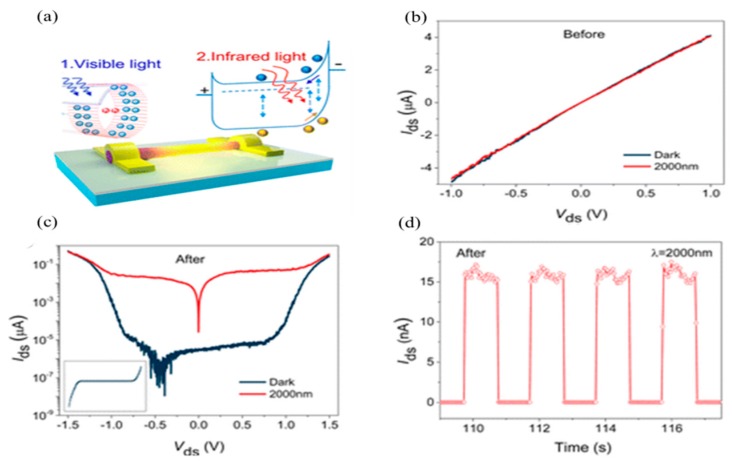
The reverse photo-response triggered IR detection in an InAs nanowire-based photodetector. (**a**) The single InAs nanowire with photo-gating mechanism under shorter wavelength illumination; (**b**) the photo-response before and (**c**) after shedding of light; (**d**) the photo-response measurement after optical activation indicating fast response speed about 100 µs in the device, (reproduced with the permission of Reference [38]. Copyright 2016, American Chemical Society).

A reverse photo-response effect in an InAs nanowire-based photodetector was also observed under optical illumination [99,100]. A high detectivity of ~2 × 10^12^ Jones and a photo-response speed of ~80 µs was achieved at 2 µm in InAs NW-based photodetectors by taking advantage of such reverse photo-response phenomena [38]. The optically triggered carrier separation mechanism in InAs NW-based photodetector is shown in Figure 7a. The photocarriers were generated under illumination of a relatively shorter wavelength (~450 nm), lower than the absorption band. Meanwhile, the generated carriers were trapped in the surface native oxides, and the remaining holes were expected to combine with the electrons in the core of the NW, so that the dark current was suppressed. As shown in Figure 7c, initially there was no photo-response observed at 2 µm. Once an InAs nanowire was illuminated with a shorter wavelength light as a precursor, the dark current was suppressed due to the negative response throughout the photodetection process.

Besides semiconducting nanowire-based infrared photodetectors, single-photon detectors based on superconducting nanowires (SNSPDs) have rapidly emerged as a highly promising and new class of photon counting technology down to the near- and mid-IR ranges [101,102]. These devices offer high efficiency, provided by orders of magnitude lower energy gaps, less noise, and excellent timing resolution. Although in visible to NIR range, there exists commercially available single-photon detectors, but they also unusable due to a rapid decrease in sensitivity.

The liquid–helium cooling requirement, relatively small active area, and low sensitivity above 2 μm are the main disadvantages of conventional SNSPDs, limiting their applications especially at the near-IR. Various SNSPDs are operated in the ultra-low temperature range of 1.7–5 K, because these are based on conventional, metallic superconductors like NbN, NbTiN, Nb, or WSi [103]. 

**Figure 8:**
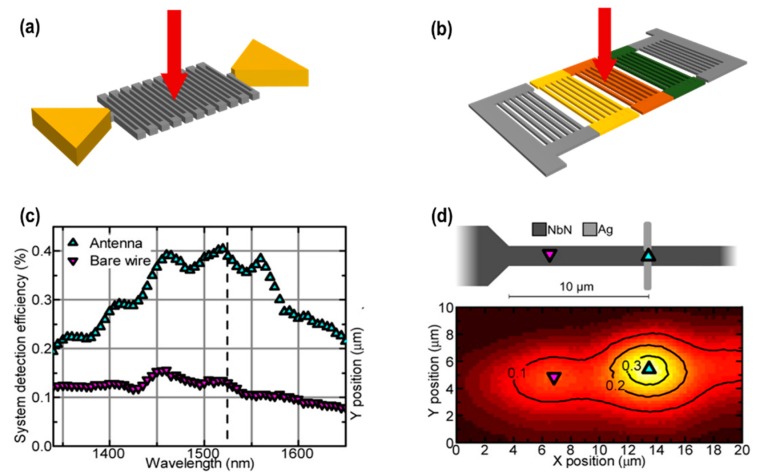
Shows the typical (**a**) meandering nanowire and (**b**) parallel-meandering nanowire single-photon detector. (**c**) The polarization at which the λ_res_ = 1525 nm nanoantenna and wire respond best are selected and (**d**) the mapping of the nanowire when the incident light is polarized with the electric field, such that the so-called antenna polarization rotates to give the highest response from the antenna—the antenna’s best polarization (reproduced with the permission of Reference [113], Copyright 2015, American Chemical Society).

Superconducting nanowire single-photodetector (SNSPD) development would not be possible without the progress made over the past decade in nanotechnology, and in particular, in nanopatterning of superconducting thin films. A typical SNSPD consists of a few nanometers of a superconducting materials of thin films in sub-micrometers. In SNSPD, the energy of the absorbed photon is distributed through an avalanche-like process, creating a nonequilibrium population of quasiparticles. This quasiparticle population then disrupts the supercurrent flow, resulting eventually in a detection event. Therefore, the broadband IR photodetection in the range near- to mid-IR range has been achieved by tailoring the geometry of thin film and by using more than one thin layer of superconducting materials. After the invention of the first operational SNSPD in 2001 by Gol’tsman et al. [104], several successes have since been made in term of performance and functionalities [105,106]. By using advanced deposition techniques and device geometries, several advanced SSPDs designs (i.e., meandering-shaped SNSPD, multiple parallel-nanowire-based SNSPD, as shown in Figure 8a,b) have been brought in to optimize photodetection performance and functionalities [106,107,108,109,110]. Mattioli et al., presented a detailed review of superconducting nanowires for single photon detection particularly in the spectral range above 1 μm [111]. A significant response at 1480 nm and 1525 nm have been achieved in the two front-side-coupled silver dipole NbN nanoantenna, and 50% to 130% in the system detection efficiency was observed (as shown in Figure 8a,b). The superconducting nanowire coupled with plasmonic nanostructures offers a pathway to increase absorption, creating larger active areas and achieving more efficient detection at longer wavelengths. Furthermore, in NbN based multiple nanowires (with a width 50 nm) connected in parallel (as shown in Figure 8b) resulted in 10 times better quantum efficiency at 3.5 μm wavelength than conventional SNSPDs [112].

### 2.3. Black Phosphorus, Graphene, and Heterostructures

The recently emerging 2D materials represented by graphene and black phosphorus (BP) are attracting intensive attention in IR detection technologies [6,7,114,115,116]. The thin atomic thickness of 2D materials possess strong out of plane quantum confinement, the optoelectronic properties of these materials are prone to be modified by the number of layers stacked and their stack sequence [117]. The thin atomic thickness of 2D materials give excellent tolerance to external strains, making the 2D materials especially attractive as the building blocks in flexible electronics [118,119,120]. Moreover, the electronic properties of 2D materials are found feasibly tuned by field effects and strains [119,120], which could be exploited for their engineering in photodetector devices. For infrared photodetection, graphene and black phosphorus are mostly explored because their broadband absorption to low energy infrared photons. For the construction of electronic devices, the ultrathin exfoliated 2D flakes are transferred to arbitrary substrates using various wet and dry methods [121,122].

It is found that at atomic layer thickness, graphene absorbs 2.3% of incident photons without significant wavelength dependence. In photodetectors, its absorption efficiency could be improved by stacking multilayers or by engineering graphene into other nanostructures, such as nanodisks [123,124]. With large carrier mobility (up to 2.5 × 10^5^ cm^2^ V^−1^ s^−1^) and ultrafast carrier dynamics (~ps) [125,126,127,128], graphene-based photodetectors are possible to work with at the extremely high frequency of 500 GHz [129]. In practical graphene-based devices, various strategies have been employed for effective separation of the photo-excited carrier in such short timescales, for example, metal-graphene junction, tunneling barrier junctions, and graphene homogeneous p–n junctions [6,130].

**Figure 9:**
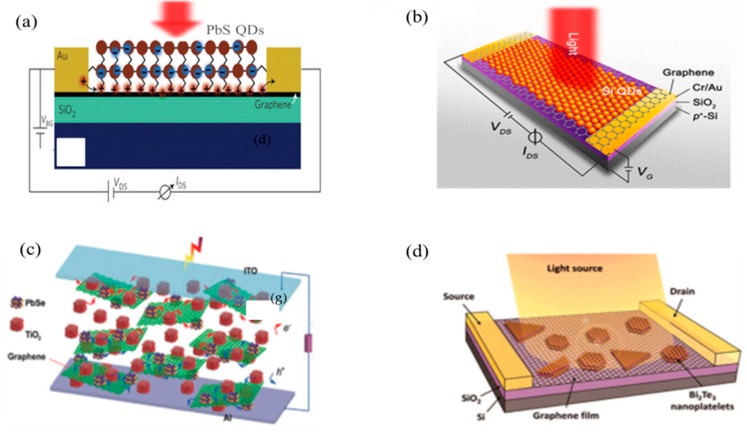
Schematics of different hybrid phototransistors based on heterostructure combinations. (**a**) PbS CQDs/graphene structure (reproduced with the permission of Reference [10]. Copyright 2012, Nature Publishing Group); (**b**) B-doped Si QD/graphene, shows the intense near-field absorption associated with the Localized Surface Plasmon Resonance (LSPR) of B-doped Si QDs (reproduced with the permission of Reference [40]. Copyright 2016, American, Chemical Society); (**c**) PbS/TiO_2_/graphene multi-interface heterostructures (reproduced with the permission of Reference [133] Copyright 2012, Wiley-VCH); (**d**) The Bi_2_Te_3_ nanoplatelets/graphene heterostructure-based photodetector (reproduced with the permission of Reference [134], Copyright 2015, American Chemical Society).

Rather than making any Schottky contacts or p–n junctions, the photogenerated carriers can be separated naturally by type-II band alignment in heterostructures. Notably, in hybrid heterostructures, comprehensive merits of individual materials integrated into a single device can be utilized to achieve optimized performance. For example, graphene possesses high electron mobility, while the overall IR absorption efficiency is much lower, whereas the PbS CQDs have a larger extinction coefficient. An ultra-high photoconductive gain (~10^8^) has been achieved by integrating graphene with CQDs into a single device [10,40]. Moreover, a unique feature in hybrid heterostructure-based photodetectors is the photogenerated carriers separated at the surface interface. The direct separation of photogenerated carriers could be realized in such kinds of device architecture due to type-II band offsets [39,131,132]. For the optical sensitization of ultrathin semiconductors (i.e., graphene, black phosphorus), several high-extinction co-efficient nanostructures (i.e., QDs, nanosheets [10,39]) as well as some organic dyes molecules have been used to achieve high-performance devices with different functionalities [133,134,135,136]. Recently, a significant photoresponsivity 10^7^ AW^−1^ in the NIR region were reported in PbS CQDs/graphene hybrid heterostructure-based photodetectors [10]. The light absorption occurs in the PbS CQD layer, and the carrier transport is taking place in the graphene layer (as shown in Figure 9a). The photogenerated carriers are expected to separate at the CQD/graphene layer interface due to band misalignment. In this case the electrons are retained in the CQD, whereas holes are injected in the graphene layer. The injected holes are combined with the electrons in the graphene layer, which results in a strong photogate effect. Therefore, a high gain (~10^8^ electrons per photon absorption) has been accomplished by modulating electrical conductance of the graphene layer.

Recently an extra-sensitive MWIR band photodetection was achieved using boron (B)-doped Si (Si:B) QDs (shown in Figure 9b) [40]. The extinction coefficient of graphene was relatively enhanced directly by the LSPR of the Si:B QDs, which was one of the unique features of this particulate structure. The electron–transition based optical absorption took place in the Si:B QDs in the ultraviolet (UV) to near-infrared (NIR) regions that additionally led to photogating in the graphene layers resulting in gain in the MIR band. Therefore, the ultra-broadband (UV–MIR) photodetection was achieved in Si:B QDs/graphene structures with a high responsivity up to ~10^9^ AW^−1^, gain up to ~10^12^, and detectivity of ~10^13^ Jones. Thus, by tuning the geometry and materials’ properties as well as the surface chemistry of QDs, a significant spectral response from shorter to longer wavelengths was achieved. In recent years, several hybrid heterostructures have been developed, like Ge/graphene [137], PbSe/MoS_2_ [132], and HgTe/MoS_2_ [138], owning significantly high responsivity in the range of visible to different IR bands. Besides single heterojunction structures, heterostructures with multiple interfaces are now prevalent due to their high detectivity and responsivity in the NIR spectrum [41]. Recently, the TiO_2_/CQDs/graphene based heterostructure has also been investigated (Figure 8c) [133]. Such kinds of structure show a high detectivity reaching up to 10^13^ Jones in the NIR region, but still keep a fast response in a few microseconds. Therefore, from such performance-related developments, it can be anticipated that there will be a remarkable potential to optimize the performance of hybrid heterostructures. The synergistically engineered heterostructures after a comprehensive understanding of optical and electrical properties of materials would provide leverage for photodetection performance in different IR bands.

### 2.4. Plasmonic Nanostructures and Related Architectures

Over the last two decades, continuously researches have been carried out about light-matter interaction, particularly in nanostructured photodetection devices. The photodetection performance has been seemingly approaching the intrinsic performance limitation of materials. Therefore, the plasmonic nanostructures and plasmonic meta-surfaces are now attracting much attention to prospective photo-detection technologies. Surface plasmon interactions can typically be divided into (i) localized surface plasmons (SPs) and (ii) surface plasmon-polaritons (SPPs). For both, the incident light wave is coupled with charge carriers in the metal by EM field, and this belted oscillation takes place at the metal/dielectric interface. This optically executed surface plasmon resonance (SPR) exponentially decayed in dielectric media, wherein the light absorption takes place [25,139,140]. The surface plasmons decay along the interface is exhibiting the light waveguide pathway [141]. Such propagating SPPs optically resonated with similar frequency. The resonance frequency depends on the geometrical structure and surrounding environment of the plasmonic nanostructure [142,143]. This unique feature of plasmonic nanostructures would provide a distinctive perspective in optoelectronic and photovoltaic devices [144,145]. Now several fabrication methods are possible for precise design of plasmonic nanostructures, such as lithography, ion beam milling, chemical synthesis routes and nano-imprinting [146].

**Figure 10:**
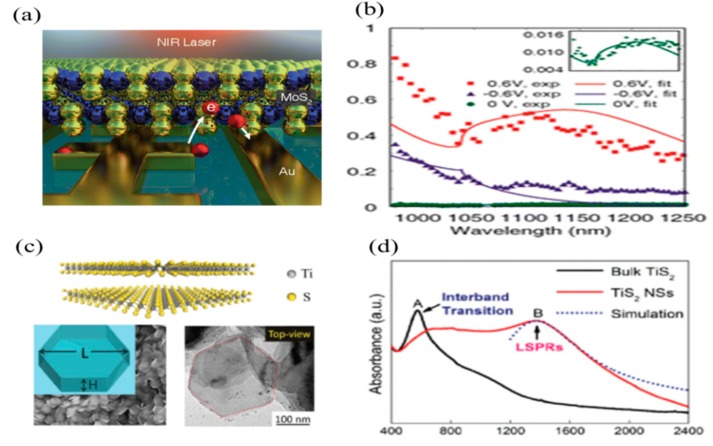
(**a**) The schematic of Hot Electron-Based Near-Infrared Photodetection Using Bilayer MoS_2_; (**b**) the photo-responsivity of the photodetector in the NIR-band under different biased voltages (Reproduced with the permission of Reference [147]. Copyright 2015, American Chemical Society); (**c**) the schematic illustration and morphologies of TiS_2_-based nanoplatelets; (**d**) the SPR enhanced spectral response in the NIR-band (Reproduced with the permission of Reference [149]. Copyright 2016, Wiley-VCH).

Recently, the hot electron-based photodetection was demonstrated at sub-bandgap in semiconducting materials integrated with a nanostructured plasmonic antenna array. Under optical illumination, the hot electrons were generated from the surface plasmon decay and injected into the semiconductor layer, which resulted in photodetection with an additional bandwidth [145,146,147,148]. Figure 10a shows a photodetector based on MoS_2_ as a semiconducting layer with a nanostructured Au electrode. A Schottky barrier was developed due to a different work functions at the MoS_2_/Au interface. Upon light irradiation, hot electrons generated by SPs, came across the Schottky barrier and were absorbed in the MoS_2_ layer, which resulted in a high gain (~10^5^) in the NIR band (shown in Figure 10b). It is known that due to energy bandgap E_g_ = 1.65 eV, the photo-response was not found in MoS_2_. The absorption of light depends on external biased voltage as shown in Figure 10b. A maximum photo-response was observed in ~1.1–1.2 µm when the external bias lies within the injection and extraction range.

Beside metallic elements, several semi-metals or heavily-doped semiconductors like ITO, TiS_2_, and materials with high carrier concentrations have also been suggested for surface resonance beyond NIR [149,150]. It has been reported that the surface plasmon resonance at relatively larger NIR band edges in semi-metallic TiS_2_ with the nanodisks type of morphology could realize the surface plasmon resonance (SPR) effect in the telecommunication band ~1.5 µm [149]. Figure 10c is illustrating the schematic of the structure of TiS_2_ layer with nanodisks type structural morphology. Additionally, a broadened hump immerged in the absorption spectra (Figure 10d), indicating the plasmon resonance induced in TiS_2_ nanostructures, which resulted in a broadband photo-response up to ~2.4 µm wavelength.

It is known that the optical response in the far-field is governed by the near-field properties of the surface plasmon resonance. By utilizing the SPR feature of metal or semi-metallic nanostructures, the photo-response capabilities of photodetectors have significantly improved, and various efforts have been devoted to enhancing and guiding the far-field EM radiations at the subwavelength level [148,151]. For successful coupling of light to SPP, different strategies have been adopted such as grooved surface texture, surface gratings, and metal-disks or porous structures [148,152]. These structures have also been used to optimize absorption efficiency in CQDs and other 2D structure as discussed in an earlier section [77,153,154]. Therefore, by exploiting the SPR feature of Au layers, an exceptionally enhanced photo-response has been achieved in HgSe CQD-based photodetectors between the MWIR and LWIR spectrum [34].

**Figure 11:**
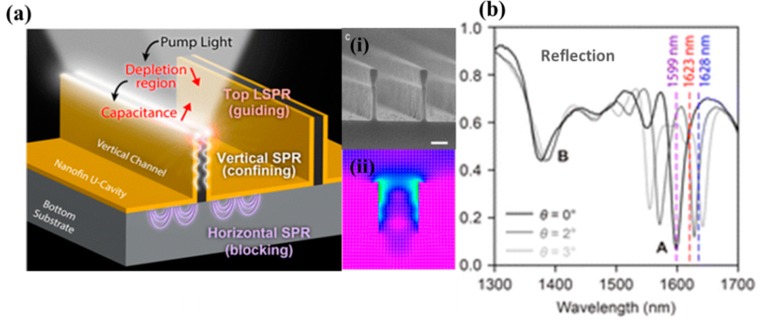
Three-dimensional plasmonic nanochannel/semiconductor structure. (**a**) Nanochannel/semiconductor with U-shaped vertical plasmonic cavities structured at semiconductor substrate, (**i**) SEM image of Si channel fin on SiO_2_ substrate, (**ii**) simulation of light concentration of EM radiations at right side; and (**b**) optical spectra showing the plasmonic coupled mode at different angles (θ) (Reproduced with the permission of [156], Copyright 2016, American Chemical Society).

Moreover, grooved surface grating and plasmonic structures provide an intense field near their peripheries which would enhance the extinction coefficient at the optical band gap edge [108,110,113]. The micro-integration of plasmonic nanostructures on the chip is difficult because of the readout in the far-field. It would be a more desirable situation to monitor the far-field signals indirectly by near-field variations. Ho et al., developed a promising strategy to focus, confine, and guide the far-field efficiently in a small volume by coupling LSPR and stationary surface plasmons [155]. Figure 10a shows the schematic of metallic U-shaped cavities, which are separated by semiconductor (Si) vertical channels, and a SEM image at the right side of Figure 10a(i) shows the cross-section of these channels on the substrate. The semiconductor channel acts as a dielectric between metallic cavities forming a capacitor-like structure. The irradiated light is focused in the channel by the LSPR at the tops of the nano-fins which are then guided and confined on the channel boundaries vertically by the SPR, which can be observed by simulation studies (as shown on the right side of Figure 11a(ii). The incident light is concentrating on the channel without much leakage and is efficiently converted into photocarriers which results in impedance modulation of the structure. The plasmonic coupled modes show narrow bandwidths and strong absorption at point A with peak wavelength centered at about 1.59 µm (Figure 11b). The more important thing here is reflectance and absorptance bandwidth, and intensity depends upon nanochannel cross-sectional width and material. Likewise, with various particular designs of a metal coupler, spectral selectivity has been transformed into polarization and color detection [148,152,156].

## 3. Summary

**Figure 12:**
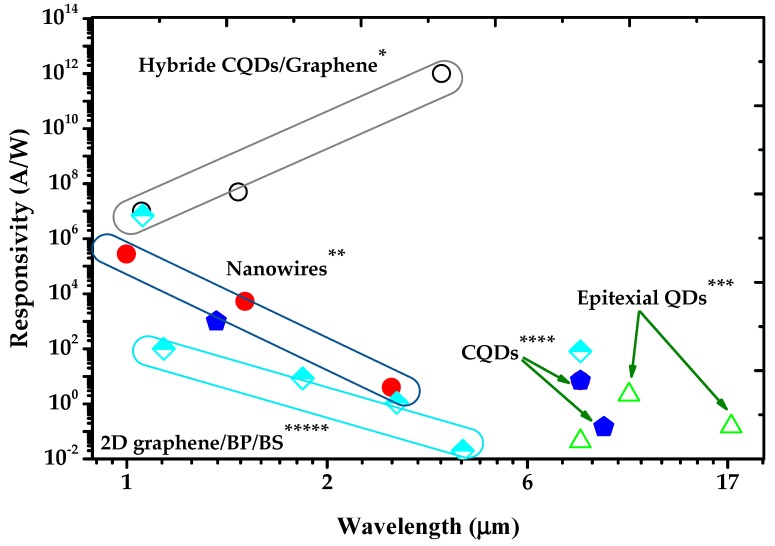
A summary of the responsivity of infrared photodetectors based on different nanostructured materials versus the detection wavelength [10,39,40,41] *; [36,37,38] **; [28,29,30,31] ***; [33,34,35] ****; [153,157,158,159] *****.

In this review, we have briefly surveyed recent developments in the field of IRPDs based on different nanostructured materials which are considered essential building blocks due to various exclusive optoelectronic properties associated with their geometrical structures. We have also observed several achievements in the performance and functionalities of IRPDs regarding detectivity, responsivity, and polarization sensitivity. Such achievements are realized with optimization of carrier dynamics in nanostructured materials by feasible tailoring of the optical band gap of quantized systems, and nano-fabrication of polarization sensitive anisotropic materials and architectures. In Figure 12, responsivity values of the representative nanostructured materials are selected from Table 1 and plotted versus their detection wavelengths. Due to the broadband nature of infrared spectra, there are hardly materials that could fulfil all the requirements of IR detection from NIR to FIR. As displayed in the figures, the nanostructured materials represented by quantum dots, nanowires, and hybrid materials are showing unprecedented high performances in the NIR to SWIR bands. This is benefited from the feasible engineering of photoconductive gain mechanisms, e.g., by surface tailoring, field effect modulations or building heterojunctions.

Although several great successes have been achieved, some challenges are still in the way to achieve optimized performance in IRPDs, like the paradoxical relation between optical gain and response time, and wavelength detection limit and operational temperature. However, the hybrid heterostructure combinations are likely to play essential roles in perspective high-performance IRPDs with regards to the challenges discussed. Moreover, the integration of traditional low-dimensional nanostructured materials with plasmonic nanostructures and plasmonic waveguides is crucial for the construction of photodetectors beyond the optical detection limit of a particular material. Therefore, considering the significant achievements in nanotechnology with a variety of materials, we are expecting the bright future of IRPDs based on nanostructured materials.

## Figures and Tables

**Table 1:** The list of nanostructured materials and their performance parameters for IR detectors.

Category	Materials	Wavelength (µm)	Responsivity [AW^−1^]	Detectivity [Jones]	Speed [Hz, s]	Pub. Year	Ref.
Epitaxially Grown QDs	InGaAs/GaAs/AlGaAs	17	0.15	1.0 × 10^7^	--	2007	[28]
	GaSb/AlSb	3.0	--	1.8 × 10^11^	--	2013	[29]
	GaAs/AlGaAs	8.0	0.044	4.5 × 10^11^	--	2005	[30]
	InGaAs/GaAs	10.2	2.16	1.0 × 10^11^	--	2011	[31]
Quantum wells	InGaAs/InP	8.0	7.0	7.4 × 10^10^	--	2010	[22]
Colloidal QDs	HgTe QDs	2–65	--	--	--	2018	[32]
	HgTe QDs	2.0	--	2 × 10^10^	2 KHz	2017	[33]
	HgSe QDs	4.2–9	0.145	--	--	2017	[34]
	PbS QDs	1.3	1.0 × 10^3^	1.8 × 10^13^	18 Hz	2006	[35]
Nanowires	InP	0.83	2.8 × 10^5^	9.1 × 10^15^	139 ms	2016	[36]
	InAs	1.5	5.3 × 10^3^	--	--	2014	[37]
	InAs	2–3	4.0–0.6	2.0 × 10^12^	≈0.1 ms	2016	[38]
Hybrid Heterostructures	PbS QDs/Gr	0.895	1.0 × 10^7^	--	0.3–1.7s	2012	[39]
	PbS QDs/Gr	0.6–1.4	5 × 10^7^–5 × 10^5^	7.0 × 10^13^	--	2012	[10]
	B-Si QDs/Gr	0.4–4	~10^12^	~10^13^	--	2017	[40]
	Perovskite/TiO2/Si	1.1	0.87	6 × 10^12^	--	2017	[41]
